# Screening Cutoff Values for the Detection of Aldosterone-Producing Adenoma by LC-MS/MS and a Novel Noncompetitive CLEIA

**DOI:** 10.1210/jendso/bvae080

**Published:** 2024-04-20

**Authors:** Yoshikiyo Ono, Yuta Tezuka, Kei Omata, Ryo Morimoto, Yuto Yamazaki, Sota Oguro, Kei Takase, Akihiro Ito, Tatsunari Yoshimi, Satoshi Kojima, Sadayoshi Ito, Hironobu Sasano, Takashi Suzuki, Tetsuhiro Tanaka, Hideki Katagiri, Fumitoshi Satoh

**Affiliations:** Department of Diabetes, Metabolism and Endocrinology, Tohoku University Hospital, Sendai, Miyagi 980-8574, Japan; Division of Nephrology, Rheumatology and Endocrinology, Tohoku University Graduate School of Medicine, Sendai, Miyagi 980-8575, Japan; Department of Diabetes, Metabolism and Endocrinology, Tohoku University Hospital, Sendai, Miyagi 980-8574, Japan; Division of Nephrology, Rheumatology and Endocrinology, Tohoku University Graduate School of Medicine, Sendai, Miyagi 980-8575, Japan; Department of Diabetes, Metabolism and Endocrinology, Tohoku University Hospital, Sendai, Miyagi 980-8574, Japan; Division of Nephrology, Rheumatology and Endocrinology, Tohoku University Graduate School of Medicine, Sendai, Miyagi 980-8575, Japan; Division of Nephrology, Rheumatology and Endocrinology, Tohoku University Graduate School of Medicine, Sendai, Miyagi 980-8575, Japan; Department of Pathology, Tohoku University Graduate School of Medicine, Sendai, Miyagi 980-8575, Japan; Department of Diagnostic Radiology, Tohoku University Graduate School of Medicine, Sendai, Miyagi 980-8575, Japan; Department of Diagnostic Radiology, Tohoku University Graduate School of Medicine, Sendai, Miyagi 980-8575, Japan; Department of Urology, Tohoku University Graduate School of Medicine, Sendai, Miyagi 980-8575, Japan; Research and Development Division, Fujirebio Inc, Hachioji, Tokyo 192-0031, Japan; Research and Development Division, Fujirebio Inc, Hachioji, Tokyo 192-0031, Japan; Division of Nephrology, Rheumatology and Endocrinology, Tohoku University Graduate School of Medicine, Sendai, Miyagi 980-8575, Japan; Department of Pathology, Tohoku University Graduate School of Medicine, Sendai, Miyagi 980-8575, Japan; Department of Pathology, Tohoku University Graduate School of Medicine, Sendai, Miyagi 980-8575, Japan; Division of Nephrology, Rheumatology and Endocrinology, Tohoku University Graduate School of Medicine, Sendai, Miyagi 980-8575, Japan; Department of Diabetes, Metabolism and Endocrinology, Tohoku University Hospital, Sendai, Miyagi 980-8574, Japan; Division of Nephrology, Rheumatology and Endocrinology, Tohoku University Graduate School of Medicine, Sendai, Miyagi 980-8575, Japan; Department of Pathology, Tohoku University Graduate School of Medicine, Sendai, Miyagi 980-8575, Japan

**Keywords:** primary aldosteronism, aldosterone, assay, screening, diagnosis

## Abstract

**Context:**

Detecting patients with surgically curable aldosterone-producing adenoma (APA) among hypertensive individuals is clinically pivotal. Liquid chromatography–tandem mass spectrometry (LC-MS/MS) is the ideal method of measuring plasma aldosterone concentration (PAC) because of the inaccuracy of conventional chemiluminescent enzyme immunoassay (CLEIA). However, LC-MS/MS is expensive and requires expertise. We have developed a novel noncompetitive CLEIA (NC-CLEIA) for measuring PAC in 30 minutes.

**Objective:**

This work aimed to validate NC-CLEIA PAC measurements by comparing them with LC-MS/MS measurements and determining screening cutoffs for both measurements detecting APA.

**Methods:**

We retrospectively measured PAC using LC-MS/MS and NC-CLEIA in 133 patients with APA, 100 with bilateral hyperaldosteronism, and 111 with essential hypertension to explore the accuracy of NC-CLEIA PAC measurements by comparing with LC-MS/MS measurements and determined the cutoffs for detecting APA.

**Results:**

Passing-Bablok analysis revealed that the values by NC-CLEIA (the regression slope, intercept, and correlation coefficient were 0.962, −0.043, and 0.994, respectively) were significantly correlated and equivalent to those by LC-MS/MS. Bland-Altman plot analysis of NC-CLEIA and LC-MS/MS also demonstrated smaller systemic errors (a bias of −0.348 ng/dL with limits of agreement of −4.390 and 3.694 within a 95% CI) in NC-CLEIA than LC-MS/MS. The receiver operating characteristic analysis demonstrated that cutoff values for aldosterone/renin activity ratio obtained by LC-MS/MS and NC-CLEIA were 31.2 and 31.5 (ng/dL per ng/mL/hour), with a sensitivity of 91.0% and 90.2% and specificity of 75.4% and 76.8%, respectively, to differentiate APA from non-APA.

**Conclusion:**

This newly developed NC-CLEIA for measuring PAC could serve as a clinically reliable alternative to LC-MS/MS.

Primary aldosteronism (PA) is one of the most frequent causes of secondary hypertension, and hypertension caused by unilateral PA is generally curable or at least can be improved after surgical treatment [1-6]. It has become pivotal for clinicians to detect surgically curable PA among hypertensive individuals. The Endocrine Society recommends measuring plasma aldosterone concentration (PAC) and plasma renin activity (PRA) or active renin concentration and calculation of the aldosterone-to-renin ratio (ARR) to screen hypertensive patients for the clinical detection of PA [1]. The diagnostic workup of PA also includes confirmatory testing by saline infusion, captopril challenge, or fludrocortisone suppression, and determining the PA subtypes according to adrenal computed tomography and adrenal venous sampling (AVS) [1-6]. Accurate and reproducible measurement of peripheral PAC is generally considered pivotal in all diagnostic workups of patients with PA regardless of their clinical stages. Peripheral aldosterone concentrations usually varies from 1 to 100 ng/dL, and because of these relatively low values, highly sensitive and specific assays are mandatory for the accurate measurement of PAC. However, for the last several decades, PAC has been mainly measured by antibody-based immunoassays that did not necessarily harbor high specificity and more than frequently resulted in overestimation of PAC, in addition to marked variability in assay performance reported among different laboratories [7, 8]. Therefore, to overcome those difficulties in measuring PAC, liquid chromatography–tandem mass spectrometry (LC-MS/MS) was applied. LC-MS/MS was reported to be more reliable than conventional radioimmunoassay (RIA) and, therefore, has been employed in some institutions in which many patients with PA were managed [9-12]. However, LC-MS/MS requires more expensive and expertise, which has unfortunately prevented its widescale application in routine clinical practice. Therefore, to circumvent these pitfalls of LC-MS/MS for measuring PAC, we and others recently developed a fully automated chemiluminescent enzyme immunoassay (CLEIA) [7, 13]. CLEIA has markedly contributed to shortening the workup time of patients with PA, because almost all CLEIAs of PAC have used conventional competition curve analysis as in RIA. However, it is also true that a conventional CLEIA was not necessarily associated with high specificity and sensitivity compared with LC-MS/MS analysis [7, 13]. Therefore, many clinical laboratories still use LC-MS/MS for the accurate analysis of PAC. LC-MS/MS could specifically measure lower values of PAC compared to RIA or CLEIA, thus establishing that method-specific cutoff values for PA screening and confirmatory testing is therefore required [9-12, 14]. Several Australian and German research groups very recently independently confirmed the approximately 20% to 50% lower values of PAC measured by LC-MS/MS, and proposed the new cutoff values toward the clinical diagnosis of PA compared with PAC measured by RIA or CLEIA [9-11, 14]. These differences, especially at lower concentrations (<10 ng/dL), could be critical for diagnosing or screening for PA, because these cutoff values of both baseline and confirmatory tests for PAC including ARR component could be at a lower concentration [1, 9-11, 14].

The SPAC-S aldosterone kit (SPAC) (Fujirebio Inc) has been used for analyzing PAC in almost all clinical laboratories since 2009 in Japan, when the Japanese Guidelines 2009 for diagnosis and treatment of PA were formulated [4]. However, the SPAC was commercially terminated in March 2021. We recognized that SPAC also had limitations as in other RIAs compared to LC-MS/MS. On the other hand, we developed the LC-MS/MS method for PAC with Aska Pharma Medical Co. Ltd. in 2008 [15]. Since then, we have used the standardized PAC values by LC-MS/MS for the diagnosis of PA [13, 16]. We recently developed a noncompetitive (NC-) CLEIA for PAC (a sandwich method of 2 antibodies), which is closely equivalent to LC-MS/MS, in conjunction with Fujirebio Inc. This novel assay could swiftly provide results in approximately 30 minutes and has been approved and covered by the universal health insurance system of the Japanese government.

Therefore, in our present study, we further validated this NC-CLEIA PAC and compared the results obtained with those by LC-MS/MS, a gold standard of measurement. We then studied its clinically validated diagnostic capacity to differentiate between patients with surgically curable aldosterone-producing adenoma (APA) and non-APA patients, including those with bilateral hyperaldosteronism (BHA) and essential hypertension (EH). These validations were all performed in the plasma samples of the patients cryopreserved after the pathological diagnosis of APA.

## Materials and Methods

### Patient Eligibility and Diagnosis of Primary Aldosteronism

We screened hypertensive patients, including those with PA, who visited Tohoku University Hospital between December 2016 and September 2019, as previously reported [13]. We measured PAC and plasma renin activity using conventional RIA kits to clinically determine the ARR. At this point, we obtained residual blood samples from the patients accompanied by research information disclosure. Following centrifugation, the plasma samples were immediately frozen and stored at −20 °C for future aldosterone measurement.

If the patients showed ARR values by RIA equal to or more than 20 ng/dL per ng/mL/h, they proceeded to undergo a captopril challenge test for diagnosing PA or EH as the Japanese guideline recommends [4]. Our criterion for diagnosing PA was based on ARRs surpassing 20 ng/dL per ng/mL/h after being captopril challenged. Those who were confirmed to be clinically negative for PA were additionally screened for other etiologies of secondary hypertension, such as Cushing syndrome, pheochromocytoma, and renal artery stenosis, were excluded, and finally diagnosed to have EH. We performed segmental AVS, which can accurately detect PA subtypes such as APA, which is curable by surgery, and BHA (Supplementary Fig. S1) [6, 17-19]. Pathological study was performed to confirm the presence of aldosterone-producing lesions including immunohistochemistry of CYP11B2 (RRID:AB_2650562), as reported previously [20-24]. Eligible patients with PA were registered as a cohort of the PA Sendai Study with written informed consent, and the present study was approved by the ethics committee of Tohoku University School of Medicine (No. 2019-1-591).

### Conventional Radioimmunoassay of Plasma Aldosterone and Renin Activity

Plasma samples were collected in a recumbent position early in the morning after bed rest for 30 minutes. PAC was measured by RIA using the SPAC-S Aldosterone Kit (Fujirebio Inc) and PRA by RIA of angiotensin I using the Renin Activity Kit YAMASA (Yamasa Co); the analytical sensitivity was 2.5 ng/dL for PAC and 0.2 ng/mL/hour for PRA.

### Development of a Novel Chemiluminescence Assay of Plasma Aldosterone Concentration

We used the Lumipulese Presto Aldosterone (Fujirebio Inc, catalog No. 298572, RRID: AB_3076349, AB_3076350) for PAC with a fully automated, 2-step sandwich chemiluminescent enzyme immunoassay for LUMIPULSE L system (Fujirebio Inc) [25, 26]. A detailed description of the assay protocol is presented in the supplementary data [19]. The protocol is summarized briefly as follows: A total of 30 µL of plasma was mixed with 50 µL of antialdosterone monoclonal antibody–coated magnetic particle solution and incubated for 8 minutes at 37 °C (first reaction); 50 µL of alkaline phosphatase-conjugated antimetatype antibody was added and incubated for 8 minutes at 37 °C following a wash (second reaction); then, a substrate solution was added after the second reaction followed by another washing procedure to measure chemiluminescence [27]. Results were fully and automatically obtained in approximately 30 minutes (Supplementary Fig. S2) [19].

### Plasma Samples Used for Validation of the Novel Assay and Comparison to Conventional Methods

For the measurement of PAC and PRA, blood was collected from an antecubital vein into EDTA-2Na tubes in a supine position after bed rest for 30 minutes, immediately centrifuged at room temperature, and then stored at a temperature below −20 °C.

### Validation of the Novel Assay of Plasma Aldosterone

For the measurement of PAC, correlation and Passing-Bablok regression analyses and Bland-Altman plots were performed to compare the results of the novel assay with those of the LC-MS/MS method that we previously reported [15]. The same analyses were also performed to compare the results of the conventional radioimmunoassay (SPAC-S kit) with those of LC-MS/MS.

### Clinical Evaluation of Diagnostic Ability Based on the Novel Assay System

PAC and ARR determined by LC-MS/MS, NC-CLEIA, and RIA were both evaluated using receiver operating characteristics (ROC) analysis to investigate the cutoff values, sensitivity, and specificity for screening patients with APA from hypertensive patients including BHA and EH.

### Statistical Analysis

Values below a lower limit of detection were assigned to analytical sensitivity values of each assay for subsequent statistical comparison. The normality of the collected data was analyzed using the Kolmogorov-Smirnov test. When continuous variables showed a normal distribution, one-way analysis of variance was employed for comparison. Otherwise, the Kruskal-Wallis test with Dunn's multiple comparison test as a post hoc test was used. Passing-Bablok regression and Bland Altman method were used to compare the measurement values of NC-CLEIA to those of the LC-MS/MS method. The optimal cutoff value of both PAC and ARR were estimated by analyzing the ROC curve and determining the Youden index for each cutoff value. Area under curves (AUC) were also calculated for each PAC and ARR, respectively. Pairwise comparison of AUCs was used to assess AUC differences in its ability of detecting APA. Statistical significance was set at *P* less than .05, and statistical analysis was performed using Stat Flex software (version 7.1; Artech Co Ltd).

## Results

### Validation of the Novel Assays for Plasma Aldosterone Concentrations

The detailed results of the validation experiments are presented in the supplementary data [19]. Briefly, for the PAC assay, the limit of quantification at 10% coefficient of variation was 0.22 ng/dL, as shown in Supplementary Table S1 and Supplementary Fig. S3 [19]. Assay ranges (defined as analytical sensitivity to upper limit of detection) were set to 0.2 to 180.0 ng/dL for the PAC assay. Precision results are presented in Supplementary Table S2 for the PAC assay [19]. Linearity results of the PAC assay are shown in Supplementary Table S3 [19]. Detailed results of spike recovery, endogenous interference, therapeutic and drug interference, and cross-reactivity for the PAC assay are shown in Supplementary Tables S4, S5, S6, and S7, respectively [19].

### Diagnosis of Primary Aldosteronism and Essential Hypertension

Endocrinological diagnosis was performed using the conventional SPAC-S Kit for PAC and Renin Activity Kit YAMASA for PRA. A total of 344 patients were judged as eligible for the present study, and of them, 233 had PA. Among these 233 patients, 133 (57%) were diagnosed to have unilateral disease by segmental AVS; after adrenalectomy, they were finally diagnosed with APA (Table 1). A total of 100 patients (43%) were diagnosed with BHA and the remaining 111 patients were diagnosed with EH (see Table 1). Based on the results obtained by conventional RIA PAC, ARR, and captopril-challenged ARR were significantly higher in those with APA than in those with BHA or EH (*P* < .05). PRA was significantly higher in those with EH than in those with APA or BHA (*P* < .05), while comparison between those with APA and BHA showed no significant difference in baseline PRA levels.

**Table 1.** Clinical characteristics of the aldosterone-producing adenoma, bilateral hyperaldosteronism, and essential hypertension groups

	APA	BHA	EH	*P*
No.	133	100	111	
Age, y	51.7 ± 12.7	56.7 ± 10.1	49.0 ± 14.6	<.05*^ab^*
Sex, male, female	75, 58	33, 67	45, 66	
Body mass index	25.1 ± 4.8	24.8 ± 4.1	26.0 ± 5.6	NS
Systolic blood pressure, mm Hg	148.4 ± 17.0	145.8 ± 17.5	144.1 ± 25.6	NS
Diastolic blood pressure, mm Hg	95.4 ± 10.3	93.6 ± 11.5	93.1 ± 19.0	NS
Heart rate, beat per min	75.7 ± 13.0	76.9 ± 10.8	80.1 ± 13.3	<.05*^c^*
No. of antihypertensive agents per d	1.9 ± 1.3	1.6 ± 1.0	1.1 ± 0.9	<.05*^c^*
eGFR, mL/min/1.73m^2^	79.4 ± 28.5	77.0 ± 18.1	86.3 ± 28.6	NS
Serum potassium, mM	3.4 ± 0.5	3.9 ± 0.5	4.0 ± 0.4	<.05*^ac^*
Potassium replacement, mmol/d	71.4 ± 42.2	18.3 ± 26.0	9.2 ± 17.9	<.05*^abc^*
LC-MS/MS PAC, ng/dL	32.5 ± 22.1	8.5 ± 6.1	8.6 ± 6.9	<.05*^ac^*
NC-CLEIA PAC, ng/dL	32.1 ± 21.0	8.3 ± 5.7	8.2 ± 6.5	<.05*^ac^*
RIA PAC, ng/dL	48.3 ± 25.9	17.6 ± 8.7	17.9 ± 10.1	<.05*^ac^*
Plasma renin activity, ng/mL/h	0.3 ± 0.2	0.3 ± 0.1	1.1 ± 1.0	<.05*^bc^*
ARR with LC-MS/MS PAC, ng/dL per ng/mL/h	133.7 ± 114.4	34.5 ± 23.4	13.4 ± 12.2	<.05*^abc^*
ARR with NC-CLEIA PAC, ng/dL per ng/mL/h	131.8 ± 109.4	33.8 ± 22.1	12.6 ± 11.2	<.05*^abc^*
ARR with RIA PAC, ng/dL per ng/mL/h	197.7 ± 140.5	73.0 ± 37.8	28.3 ± 20.0	<.05*^abc^*
Captopril-challenged ARR with RIA PAC, ng/dL per ng/mL/h	225.3 ± 250.7	53.1 ± 30.7	11.0 ± 5.1	<.05*^abc^*
Cortisol with 1 mg dexamethasone suppression test, µg/dL	1.2 ± 0.9	0.9 ± 0.5	0.9 ± 0.6	<.05*^ac^*

Data are shown as mean ± SD.

Abbreviations: APA, aldosterone-producing adenoma; ARR, aldosterone-to-renin activity ratio; BHA, bilateral hyperaldosteronism; EH, essential hypertension; eGFR, estimated glomerular filtration rate; LC-MS/MS, liquid chromatography–tandem mass spectrometry; NC-CLEIA, noncompetitive-chemiluminescent enzyme immunoassay; NS, not significant; PAC, plasma aldosterone concentration; RIA, radioimmunoassay.

^*a*^Denotes statistical significance in comparison between APA and BHA groups.

^*b*^Denotes statistical significance in comparison between BHA and EH groups.

^*c*^Denotes statistical significance in comparison between APA and EH groups.

### Validation of the Novel Assay of Plasma Aldosterone Concentration

**Figure 1.**
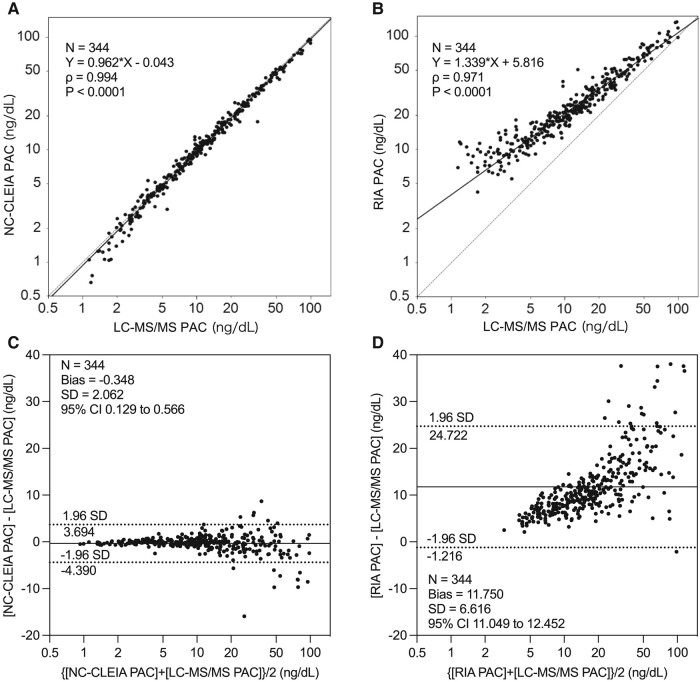
Correlation of plasma aldosterone concentration (PAC) between A, noncompetitive chemiluminescent enzyme immunoassay (NC-CLEIA) and liquid chromatography–tandem mass spectrometry (LC-MS/MS) and correlation of PAC between B, radioimmunoassay (RIA) and LC-MS/MS. Bland-Altman plot analysis of PAC C, between LC-MS/MS and NC-CLEIA and D, between LC-MS/MS and RIA. In the Bland-Altman plot analysis, solid line indicates mean difference (bias), and dotted lines indicate 95% limits of agreement, respectively.

PAC were measured using NC-CLEIA in all eligible patients. PAC by NC-CLEIA were significantly higher in those with APA when compared to those with BHA or EH (see Table 1). When the BHA subgroup was compared with the EH subgroup, PAC by NC-CLEIA was significantly elevated in those with BHA (see Table 1). The measurements of PAC in all specimens measured by NC-CLEIA were significantly correlated with those obtained by LC-MS/MS (Spearman correlation coefficient [ρ] = 0.994; *P* < .0001) with a Passing-Bablok regression model of Y = 0.962X-0.043 (Fig. 1A). The concentration gradient of the correlation was almost one, and there was almost none in the graft. Measurements of PAC by NC-CLEIA in the specimens with less than 10 ng/dL and 10 ng/dL or higher PAC by LC-MS/MS were significantly correlated with those of LC-MS/MS (ρ = 0.969; *P* < .0001 and ρ = 0.991; *P* < .0001, respectively) with a Passing-Bablok model of Y = 1.021X-0.301 (Supplementary Fig. S4A) and Y = 0.950X + 0.205 (Supplementary Fig. S4B), respectively [19]. These inclines of the correlations in both ranges of PAC hardly differed. Measurements of PAC in all specimens using the conventional RIA SPAC-S Kit were also correlated with those of LC-MS/MS (ρ = 0.971; *P* < .0001), with a Passing-Bablok model of Y = 1.339X + 5.816 (Fig. 1B). As compared with the former, the correlation of RIA and LC-MS/MS showed a large graft, and the incline of the correlation was bigger as well. Measurements of PAC by RIA in the specimens with less than 10 ng/dL and 10 ng/dL or higher PAC by LC-MS/MS were significantly correlated with those of LC-MS/MS (ρ = 0.781; *P* < .0001 and ρ = 0.962; *P* < .0001, respectively), with a Passing-Bablok model of Y = 1.836X + 3.467 (Supplementary Fig. S4C) and Y = 1.285X + 6.589 (Supplementary Fig. S4D), respectively [19]. Unlike the former, the inclines of the correlations were changed at different ranges of PAC. Comparison of NC-CLEIA PAC with RIA of PAC yielded results similar to that between LC-MS/MS PAC and RIA of PAC (Supplementary Fig. S6A-S6C) [19].

Bland-Altman plot analysis of PAC in all specimens by NC-CLEIA and LC-MS/MS revealed a bias of −0.348 ng/dL, and the limits of agreement were −4.390 and 3.694 with 95% CI (Fig. 1C). The comparison between NC-CLEIA and LC-MS/MS for specimens with less than 10 ng/dL of PAC by LC-MS/MS showed a bias of −0.018 ng/dL and limits of agreement of −1.402 and 1.366 with 95% CI (Supplementary Fig. S5A), whereas for specimens with 10 ng/dL or higher of PAC, LC-MS/MS revealed a bias of −0.612 ng/dL with limits of agreement of −5.842 and 4.619 with 95% CI (Supplementary Fig. S5B) [19]. These biases were astonishingly small, suggesting that the systemic errors of this assay were exceedingly small, especially in the lower range of PAC. On the other hand, Bland-Altman plot analysis of PAC in all specimens by RIA and LC-MS/MS revealed a larger bias of 11.750 ng/dL, and the limits of agreement were −1.216 and 24.72 with 95% CI (Fig. 1D). The comparison between PAC by RIA and LC-MS/MS in specimens with less than 10 ng/dL of PAC by LC-MS/MS showed a bias of 8.131 ng/dL and limits of agreement of 1.270 and 14.993 with 95% CI (Supplementary Fig. S5C), whereas for specimens with 10 ng/dL or higher of PAC by LC-MS/MS, the bias was 14.649 ng/dL and the limits of agreement were 0.760 and 28.537 with 95% CI (Supplementary Fig. S5D) [19]. Unlike the former, the biases were large, suggesting greater systemic errors for RIA, even in the lower range of PAC. Considering the aforementioned results, the accuracy of NC-CLEIA was quite superior to that of the RIA, especially when PAC was less than 10 ng/dL, which is a critical level for screening and confirming PA. Comparison of NC-CLEIA PAC with RIA of PAC demonstrated results similar to that between LC-MS/MS PAC and RIA of PAC (Supplementary Fig. S6D-S6F) [19].

### Screening Ability of Aldosterone-Producing Adenoma Based on the Novel Assay of Plasma Aldosterone Concentration

**Figure 2.**
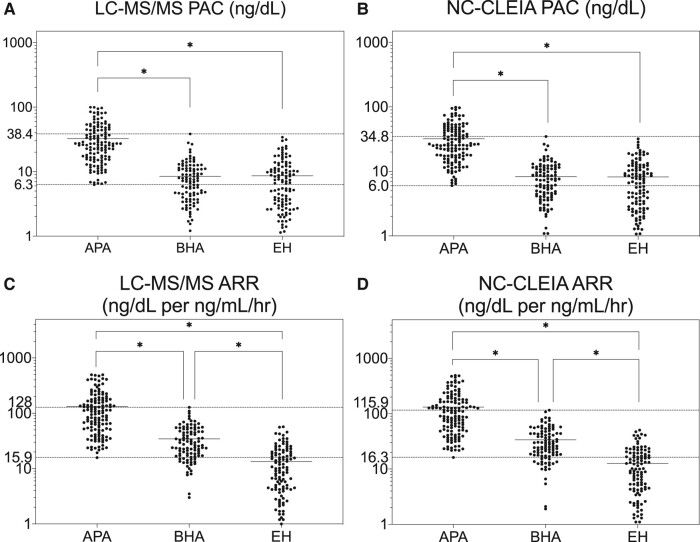
Distribution of A, liquid chromatography–tandem mass spectrometry (LC-MS/MS) and B, noncompetitive chemiluminescent enzyme immunoassay (NC-CLEIA) of plasma aldosterone concentration (PAC) measurements in aldosterone-producing adenoma (APA), bilateral hyperaldosteronism (BHA), and essential hypertension (EH). Distribution of C, LC-MS/MS and D, NC-CLEIA of aldosterone/renin activity ratio (ARR) in APA, BHA, and EH. *Indicates *P* less than .05.

Baseline levels of PAC and ARR by LC-MS/MS, NC-CLEAR, and RIA are summarized in Table 1, Fig. 2, and Supplementary Fig. S5, respectively [19]. Values of ARR by LC-MS/MS, NC-CLEIA, and RIA were significantly higher in those with APA when compared with those with BHA or EH (see Table 1; Figs. 2C and 2D, and Supplementary Fig. S7B) [19]. The lowest values of PAC in APA were 6.3, 6.0, and 12.6 ng/dL by LC-MS/MS, NC-CLEIA, and RIA, respectively (Supplementary Table S8; Fig. 2A and 2B, and Supplementary Fig. S7A) [19]. The lowest values of ARR in APA were 15.9, 16.3, and 29.6 ng/dL per ng/mL/hour by LC-MS/MS, NC-CLEIA, and RIA, respectively (see Supplementary Table S8; Figs. 2C and 2D, and Supplementary Fig. S7B) [19].

If we set PAC at 6.0 ng/dL or greater and ARR at 15 ng/dL or greater per ng/mL/hour as screening cutoff values of PA by LC-MS/MS and NC-CLEIA, the sensitivity for APA is 100% by both, and the specificity is 58.8% and 59.2%, respectively. With either method, 44.0% of BHA patients and 72.1% and 73.0%, respectively, of EH patients would come off from the workup of PA, and would be targeted for antihypertensive treatment with drugs that would include mineralocorticoid receptor blockers, or not (Supplementary Fig. S8) [19].

**Figure 3.**
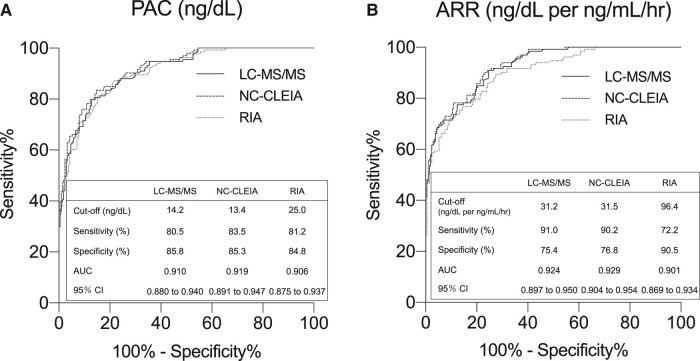
A, Receiver operating characteristics (ROC) analysis with liquid chromatography–tandem mass spectrometry (LC-MS/MS), noncompetitive chemiluminescent enzyme immunoassay (NC-CLEIA), and radioimmunoassay (RIA) of plasma aldosterone concentration (PAC) measurements to discriminate aldosterone-producing adenoma (APA) from non-APA. B, ROC analysis with LC-MS/MS, NC-CLEIA, and RIA of aldosterone/renin activity ratio (ARR) to discriminate APA from non-APA.

Based on the measurements of PAC and ARR, ROC analysis was performed to investigate the diagnostic ability of PAC and ARR as a screening test for APA among hypertensive patients, including those with BHA or EH. The ROC plot revealed that PAC by LC-MS/MS had an AUC of 0.910 (95% CI, 0.880-0.940). It demonstrated a sensitivity of 80.5% and specificity of 85.8% at a cutoff of 14.2 ng/dL (Fig. 3A, Supplementary Table S9) [19]. PAC by NC-CLEIA demonstrated an AUC of 0.919 (95% CI, 0.891-0.947), with a sensitivity of 83.5% and specificity of 85.3% at a cutoff of 13.4 ng/dL (see Fig. 3A, Supplementary Table S10) [19]; PAC by RIA yielded an AUC of 0.906 (95% CI, 0.875-0.937) at a cutoff of 25.0 ng/dL and yielded a sensitivity of 81.2% and specificity of 84.8% (see Fig. 3A). AUC of PAC by NC-CLEIA was significantly different from that by RIA.

The ROC analysis revealed that ARR by LC-MS/MS demonstrated an AUC of 0.924 (95% CI, 0.897-0.950) at a cutoff of 31.2 ng/dL per ng/mL/hour and yielded a sensitivity of 91.0% and specificity of 75.4% to discriminate APA from non-APA (see Fig. 3B, Supplementary Table S11) [19]. ARR by NC-CLEIA demonstrated an AUC of 0.929 (95% CI, 0.904-0.954) and, at a cutoff of 31.5 ng/dL per ng/mL/hour, it yielded a sensitivity of 90.2% and specificity of 76.8% (see Fig. 3B, Supplementary Table S12) [19]. ARR by RIA yielded an AUC of 0.901 (95% CI, 0.869-0.934) at a cutoff of 96.4 ng/dL per ng/mL/hour and yielded a sensitivity of 72.2% and specificity of 90.5% (see Fig. 3B). AUC of ARR by NC-CLEIA was significantly different from that by RIA. The sensitivity and specificity of PAC and ARR values by LC-MS/MS and NC-CLEIA were almost equivalent and differed from those by RIA. Sequential results of the aforementioned ROC plot analyses are shown in the supplementary data (Supplementary Tables S9-S13) [19].

In the present study, ROC analysis of ARR demonstrated that ARR of 30 ng/dL or greater per ng/mL/hour measured by both LC-MS/MS PAC and by this new NC-CLEIA PAC could be suitable as a screening cutoff value to enable APA diagnosis with a sensitivity of 91.7% for both assays, with a specificity of 73.0% and 73.9%, respectively (Supplementary Table S11 and S12) [19]. On setting PAC at 10 ng/dL or greater and PRA at less than 1.0 ng/mL/hour as the screening cutoff value for APA diagnosis using our data, the sensitivity and specificity would have been 88.0% and 73.9% with LC-MS/MS, and those measured by NC-CLEIA would have been 85.0% and 75.8%, respectively (Supplementary Fig. S9) [19].

## Discussion

LC-MS/MS measurement of PAC is currently considered a gold-standard method to diagnose PA at many medical centers throughout the world [8-12, 14]. On the other hand, Japan's universal health insurance system does not necessarily reimburse the cost of expensive LC-MS/MS of PAC. Therefore, to our regret, RIA has been used primarily because its cost is reimbursed, even though its relatively poor sensitivity and reproducibility, especially at lower concentrations of PAC, are widely recognized [28]. Therefore, a more accurate and cost-effective aldosterone assay, which is closely equivalent to LC-MS/MS, has been in demand among Japanese clinicians managing patients with PA. In this study, we first demonstrated that PAC measured by newly developed NC-CLEIA was significantly correlated with that measured by LC-MS/MS, and that the absolute values of PAC were the same between NC-CLEIA and LC-MS/MS, whereas those obtained by RIA were much higher. In addition, Eisenhofer et al [29] reported that PAC measured by LIAISON and iSYS, which are competitive chemiluminescence assays, were significantly higher than that determined by LC-MS/MS, suggestive of the questionable validity of immunoassay-based diagnosis of PA. On the other hand, the results of our present study also demonstrated that NC-CLEIA for PAC using the LUMIPULSE L system, which is a NC-CLEIA, provided almost equal absolute value to LC-MS/MS and could yield higher sensitivity than conventional competitive CLEIAs [29, 30]. In the NC-CLEIA method, the specificity of each antibody employed could be combined using 2 different types of antibodies, which reduced the influence of cross-reactivity compared to competitive assays normally using only 1 single antibody. In addition, the 2-step immunoassay allowed a washing process after each immunoreaction, reducing the influence of various endogenous interfering substances. In competitive assays, the response of the inhibition rate was reported to be low at high concentrations, resulting in the reduced levels of precision. On the other hand, in noncompetitive assays, the signal was extended with concentration, providing relatively good precision and linearity (Supplementary Tables S2 and S3, Supplementary Fig. S2). Therefore, we believe that this assay could measure aldosterone with higher accuracy than conventional immunoassays. As a result, the NC-CLEIA method was considered to cause those differences among LUMIPULSE L, LIAISON, and iSYS systems in the value, sensitivity, and accuracy for PAC. In addition, Bland-Altman plot analysis of NC-CLEIA aldosterone measurements and LC-MS/MS measurements demonstrated much smaller biases and systemic errors, indicating that NC-CLEIA could become not only a much more accurate replacement for conventional RIA or CLEIA assays measured by competition curve, but also a clinically reliable alternative to LC-MS/MS even when PAC is less than 10 ng/dL, which is very critical for screening and confirming PA.

The Endocrine Society suggests the cutoff value of PAC determined by the saline infusion test to be 5.0 to 10 ng/dL for PA [1]. Hence, the detection of lower values of PAC is critical for the diagnosis of PA by the confirmatory tests. The results of our present study demonstrated that PAC values determined by the newly developed NC-CLEIA were closely equivalent to those obtained by LC-MS/MS, even when PAC was in the lower concentration range. Therefore, this NC-CLEIA PAC measurement, which is time-efficient, cost-effective, and less labor-intensive should be the promising alternative to LC-MS/MS for the diagnosis of PA, especially in the lower aldosterone concentration range determining the LC-MS/MS adjusted cutoff values for PA diagnosis. This method is also considered important in countries like Japan where universal health care is instituted.

The recommendation of guidelines to use ARR for detecting PA is considered clinically important, particularly in selecting medications like mineralocorticoid receptor blockers for patients. However, various studies have reported on the clinical effectiveness of surgery, especially for preventing cardiovascular events and subsequently reducing the risk of incident atrial fibrillation [1, 31, 32]. The major pathological type of unilateral PAs is APA, which is curable by adrenalectomy. Therefore, the clinical differential diagnosis of APA before adrenalectomy is considered pivotal for the management of patients with PA. AVS could clearly distinguish patients with APA from non-APA patients in clinical practice. The diagnosis by AVS has also been pathologically confirmed with immunohistochemical evaluation of CYP11B2 in adrenalectomy specimens [20-23]. It is also true that medical treatment of patients with APA for a long duration is generally considered clinically difficult because of possible cardiac or renal complications. Therefore, the screening cutoff values of PAC and ARR measured by LC-MS/MS and this new NC-CLEIA to detect APA could provide pivotal information regarding clinical differential diagnosis between patients with APA and non-APA patients in conjunction with AVS.

In the present study, ROC analysis demonstrated that the cutoff values of ARR (31.2/31.5 ng/dL per ng/mL/hour) by LC-MS/MS and NC-CLEIA were almost equivalent because they showed similar sensitivity (91.0%/90.2%) and specificity (75.4%/76.8%). Therefore, we can recommend an ARR of 30.0 ng/dL or greater per ng/mL/hour as a screening cutoff both for LC-MS/MS and NC-CLEIA, which would discriminate APA with a sensitivity of 91.7% in both assays and with a specificity of 73.0% and 73.9%, respectively (see Supplementary Tables S11 and S12) [19].

The newly revised Japanese guidelines concerning PA recommend a PAC of 6.0 ng/dL or greater and ARR of 20 ng/dL or greater per ng/mL/hour as screening cutoff values for PA with 3 NC-CLEIA methods including the assay used in the present study [5]. To our regret, there were not sufficient evident data measured by the new NC-CLEIA PAC methods equivalent to that by LC-MS/MS PAC. The results of our present study therefore could provide some evidence as to the screening cutoff values in the newly revised Japanese guidelines for the management of PA. If setting the screening cutoff values in these guidelines aimed at a higher sensitivity of catching APA, the present study demonstrated that setting the values of PAC to 6.0 ng/dL or greater and ARR to 15 ng/dL or greater per ng/mL/hour as the screening cutoff could be appropriate if measured by LC-MS/MS (sensitivity/specificity: 100%/58.8%), as well as by this new assay (sensitivity/specificity: 100%/59.2%) to detect APA.

Young [33] recommended that hypertensives with a PAC of 10 ng/dL or greater and PRA of less than 1.0 ng/mL/hour should undergo confirmatory tests for PA. To detect APA using our data, the sensitivity/specificity of these popularly used cutoff values measured by LC-MS/MS and this new assay would be 88.0%/73.9% and 85.0%/75.8%, respectively. These high sensitivity and specificity values suggest that the cutoffs were of clinical usefulness for detecting APA.

In addition, Supplementary Tables S9 to S12 of ROC analyses to determine cutoff values to detect APA could provide important information considering various clinical and social conditions, including AVS availability, health insurance, and others among different countries [19]. The newly developed NC-CLEIA could become a clinically reliable alternative to LC-MS/MS for PAC measurement.

### Limitations

Our present study has some limitations. Our institute is one of the highest-volume centers for endocrine hypertension, including PA in Japan. In general, aldosterone and renin measurements are routinely conducted for screening secondary hypertension at general practitioner offices in Japan. When the ARR of patients was found to be elevated, the physician has referred them to specialized institutions such as Tohoku University Hospital. Consequently, all the patients were enrolled from the endocrine hypertension unit of Tohoku University Hospital, which could possibly result in a bias due to difference from the actual prevalence of PA in the general hypertensive patient population. In addition, the cohort of our present study was exclusively composed of Japanese patients. This specific regional and ethnicity could also result in some selection bias. Due to the retrospective and cross-sectional nature of this study, the possibility of some human errors could not be completely ruled out, which could also advertently influence the results of retrospective studies compared to prospective studies. The patients at baseline and after captopril challenge test with an ARR under 20 ng/dL per ng/mL/hour were tentatively diagnosed as EH, which was not independently validated. Therefore, this could result in verification bias and skew estimation of sensitivities; that is, the hypertensive patients with lower values of ARR in our cohort may not necessarily represent typical APA in the general clinical arena, such as normotensive, normal level of serum potassium, and without macronodular in adrenal glands. It is therefore true that these limitations could impede the capacity to present a definitive proposal.

In addition, our present study was designed to screen for APA, and further validation through confirmatory functional tests in different groups of cohorts is definitively required for clarification.

## Conclusion

RIA and CLEIA have been the main available methods for practicing clinicians to screen patients for PA, even though these assays lack high specificity and sensitivity. LC-MS/MS is ideal for measuring PAC, but it is time-consuming, labor-intensive, and expensive. The results of our present study demonstrated that the newly developed NC-CLEIA could be as useful as LC-MS/MS to measure PAC, with high specificity and sensitivity, even at a low concentration range of PAC. In addition, the NC-CLEIA is less time-consuming and expensive than LC-MS/MS. Considering circumstances of health care insurance and medical resources in many countries, this accurate, less time-consuming, and more cost-effective new assay to measure PAC could become a clinically reliable substitute for LC-MS/MS for detecting APA in the great majority of the countries with universal health care.

## Data Availability

Original data generated and analyzed during this study are included in this published article or in the data repositories listed in “References.”

## Abbreviations

APA: aldosterone-producing adenoma
ARR: aldosterone-to-renin activity ratio
AUC: area under the curve
AVS: adrenal venous sampling
BHA: bilateral hyperaldosteronism
EH: essential hypertension
eGFR: estimated glomerular filtration rate
LC-MS/MS: liquid chromatography–tandem mass spectrometry
NC-CLEIA: noncompetitive-chemiluminescent enzyme immunoassay
PA: primary aldosteronism
PAC: plasma aldosterone concentration
PRA: plasma renin activity
RIA: radioimmunoassay
ROC: receiver operating characteristic

## References

[1] Funder JW , Carey RM, Mantero F, et al The management of primary aldosteronism: case detection, diagnosis, and treatment: an endocrine society clinical practice guideline. J Clin Endocrinol Metab. 2016;101(5):1889‐1916. 26934393 10.1210/jc.2015-4061

[2] Rossi GP , Crimì F, Rossitto G, et al Identification of surgically curable primary aldosteronism by imaging in a large, multiethnic international study. J Clin Endocrinol Metab. 2021;106(11):e4340‐e4349. 34212188 10.1210/clinem/dgab482

[3] Williams TA , Lenders JWM, Mulatero P, et al Primary Aldosteronism Surgery Outcome (PASO) investigators. Outcomes after adrenalectomy for unilateral primary aldosteronism: an international consensus on outcome measures and analysis of remission rates in an international cohort. Lancet Diabetes Endocrinol. 2017;5(9):689‐699. 28576687 10.1016/S2213-8587(17)30135-3 PMC5572673

[4] Nishikawa T , Omura M, Satoh F, et al Guidelines for the diagnosis and treatment of primary aldosteronism-The Japan Endocrine Society 2009. Endocr J. 2011;58 (9):711‐721. 21828936 10.1507/endocrj.ej11-0133

[5] Naruse M , Katabami T, Shibata H, et al Japan Endocrine Society clinical practice guideline for the diagnosis and management of primary aldosteronism 2021. Endocr J. 2022;69(4):327‐359. 35418526 10.1507/endocrj.EJ21-0508

[6] Morimoto R , Omata K, Ito S, Satoh F. Progress in the management of primary aldosteronism. Am J Hypertens. 2018;31(5):522‐531. 29534182 10.1093/ajh/hpy018 PMC5905601

[7] Manolopoulou J , Fischer E, Dietz A, et al Clinical validation for the aldosterone-to-renin ratio and aldosterone suppression testing using simultaneous fully automated chemiluminescence immunoassays. J Hypertens. 2015;33(12):2500‐2511. 26372319 10.1097/HJH.0000000000000727

[8] Brown JM , Auchus RJ, Honzel B, Luther JM, Yozamp N, Vaidya A. Recalibrating interpretations of aldosterone assays across the physiologic range: immunoassay and liquid chromatography-tandem mass spectrometry measurements under multiple controlled conditions. J Endocr Soc. 2022;6(6):bvac049. 35475027 10.1210/jendso/bvac049 PMC9032635

[9] Thuzar M , Young K, Ahmed AH, et al Diagnosis of primary aldosteronism by seated saline suppression test—variability between immunoassay and HPLC-MS/MS. J Clin Endocrinol Metab. 2020;105(3):e477‐e483. 10.1210/clinem/dgz150 31676899

[10] Guo Z , Poglitsch M, McWhinney BC, et al Aldosterone LC-MS/MS assay-specific threshold values in screening and confirmatory testing for primary aldosteronism. J Clin Endocrinol Metab. 2018;103(11):3965‐3973. 30137438 10.1210/jc.2018-01041

[11] Fries CM , Bae YJ, Rayes N, et al Prospective evaluation of aldosterone LC-MS/MS-specific cutoffs for the saline infusion test. Eur J Endocrinol. 2020;183(2):191‐201. 32460235 10.1530/EJE-20-0030

[12] Hinchliffe E , Carter S, Owen LJ, Keevil BG. Quantitation of aldosterone in human plasma by ultra high performance liquid chromatography tandem mass spectrometry. J Chromatogr B Anal Technol Biomed Life Sci. 2013;913–914:19‐23. 10.1016/j.jchromb.2012.11.013 23266360

[13] Morimoto R , Ono Y, Tezuka Y, et al Rapid screening of primary aldosteronism by a novel chemiluminescent immunoassay. Hypertension. 2017;70(2):334‐341. 28652474 10.1161/HYPERTENSIONAHA.117.09078 PMC5613948

[14] Stowasser M , Ahmed AH, Cowley D, et al Comparison of seated with recumbent saline suppression testing for the diagnosis of primary aldosteronism. J Clin Endocrinol Metab. 2018;103(11):4113‐4124. 30239841 10.1210/jc.2018-01394

[15] Yamashita K , Okuyama M, Nakagawa R, et al Development of sensitive derivatization method for aldosterone in liquid chromatography–electrospray ionization tandem mass spectrometry of corticosteroids. J Chromatogr A. 2008;1200(2):114‐121. 18561939 10.1016/j.chroma.2008.05.034

[16] Satoh F , Morimoto R, Ono Y, et al Measurement of peripheral plasma 18-oxocortisol can discriminate unilateral adenoma from bilateral diseases in patients with primary aldosteronism. Hypertension. 2015;65(5):1096‐1102. 25776074 10.1161/HYPERTENSIONAHA.114.04453 PMC4642692

[17] Satoh F , Morimoto R, Seiji K, et al Is there a role for segmental adrenal venous sampling and adrenal sparing surgery in patients with primary aldosteronism? Eur J Endocrinol. 2015;173(4):465‐477. 26194502 10.1530/EJE-14-1161

[18] Satani N , Ota H, Seiji K, et al Intra-adrenal aldosterone secretion: segmental adrenal venous sampling for localization. Radiology. 2016;278(1):265‐274. 26147784 10.1148/radiol.2015142159

[19] Ono Y , Tezuka Y, Omata K, et al Data from: “Screening Cutoff Values for the Detection of Aldosterone-Producing Adenoma by LC-MS/MS and a Novel Noncompetitive CLEIA”. Zenodo. 2024. Deposited 11 January 2024. 10.5281/zenodo.11103290 PMC11074589 38715590

[20] Yamazaki Y , Nakamura Y, Omata K, et al Histopathological classification of cross-sectional image–negative hyperaldosteronism. J Clin Endocrinol Metab. 2017;102(4):1182‐1192. 28388725 10.1210/jc.2016-2986 PMC5460723

[21] Ono Y , Nakamura Y, Maekawa T, et al Different expression of 11β-hydroxylase and aldosterone synthase between aldosterone-producing microadenomas and macroadenomas. Hypertension. 2014;64(2):438‐444. 24842915 10.1161/HYPERTENSIONAHA.113.02944 PMC5478923

[22] Ono Y , Yamazaki Y, Omata K, et al Histological characterization of aldosterone-producing adrenocortical adenomas with different somatic mutations. J Clin Endocrinol Metab. 2020;105(3):e282‐e289. 31789380 10.1210/clinem/dgz235 PMC7048684

[23] Williams TA , Gomez-Sanchez CE, Rainey WE, et al International histopathology consensus for unilateral primary aldosteronism. J Clin Endocrinol Metab. 2021;106(1):42‐54. 32717746 10.1210/clinem/dgaa484 PMC7765663

[24] RRID:AB_2650562, https://scicrunch.org/resolver/RRID:AB_2650562

[25] RRID:AB_3076349, https://scicrunch.org/resolver/RRID:AB_3076349

[26] RRID:AB_3076350, https://scicrunch.org/resolver/RRID:AB_3076350

[27] Nishizono I , Iida S, Suzuki N, et al Rapid and sensitive chemiluminescent enzyme immunoassay for measuring tumor markers. Clin Chem. 1991;37(9):1639‐1644. 1716538

[28] Nishikawa T , Satoh F, Takashi Y, et al Comparison and commutability study between standardized liquid chromatography-mass spectrometry/mass spectrometry (LC-MS/MS) and chemiluminescent enzyme immunoassay for aldosterone measurement in blood. Endocr J. 2022;69(1):45‐54. 34305069 10.1507/endocrj.EJ21-0278

[29] Eisenhofer G , Kurlbaum M, Peitzsch M, et al The saline infusion test for primary aldosteronism: implications of immunoassay inaccuracy. J Clin Endocrinol Metab. 2022;107(5):e2027‐e2036. 34963138 10.1210/clinem/dgab924 PMC9016451

[30] Fortunato A , Prontera C, Masotti S, et al State of the art of aldosterone immunoassays. A multicenter collaborative study on the behalf of the Cardiovascular Biomarkers Study Group of the Italian Section of European Society of Ligand Assay (ELAS) and Società Italiana di Biochimica Clinica (SIBIOC). Clin Chim Acta. 2015;444:106‐112. 25661092 10.1016/j.cca.2015.01.028

[31] Hundemer GL , Curhan GC, Yozamp N, Wang M, Vaidya A. Cardiometabolic outcomes and mortality in medically treated primary aldosteronism: a retrospective cohort study. Lancet Diabetes Endocrinol. 2018;6(1):51‐59. 29129576 10.1016/S2213-8587(17)30367-4 PMC5953512

[32] Rossi GP , Maiolino G, Flego A, et al Adrenalectomy lowers incident atrial fibrillation in primary aldosteronism patients at long term. Hypertension. 2018;71(4):585‐591. 29483224 10.1161/HYPERTENSIONAHA.117.10596

[33] Young WF Jr . Diagnosis and treatment of primary aldosteronism: practical clinical perspectives. J Intern Med. 2019;285(2):126‐148. 30255616 10.1111/joim.12831

